# Supplementary material for: Gut microbial taxa elevated by dietary sugar disrupt memory function
Source: Transl Psychiatry. 2021 Mar 31;11:194. doi: 10.1038/s41398-021-01309-7 (PMC8012713; doi:10.1038/s41398-021-01309-7)
Supplement: Supplementary file 3 — Supplementary Figures [file 41398_2021_1309_MOESM3_ESM.pdf]

Supplemental Figures

Figure S1

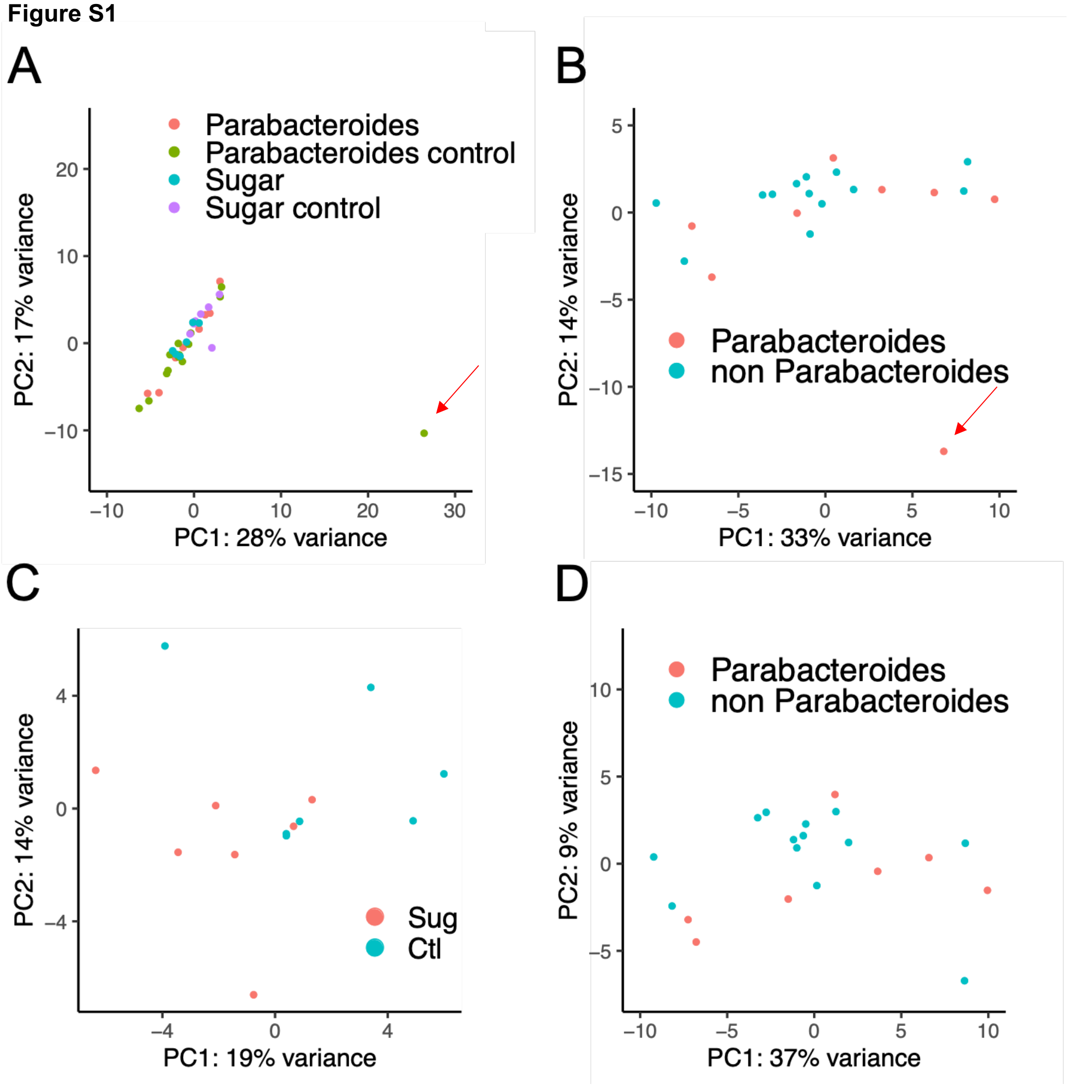

Figure S1: Principal component analyses (PCA) of hippocampal gene expression data to identify outliers (A) PCA identified one control sample (red arrow) as an outlier when all samples from both sugar and *Parabacteroides* enrichment experiments were considered. (B) PCA identified one treatment sample (red arrow) from the *Parabacteroides* experiment as an outlier. After removing the outliers, PCA for the remaining samples from the sugar treatment experiment (C) and those from the *Parabacteroides* enrichment experiments (D).

Figure S2

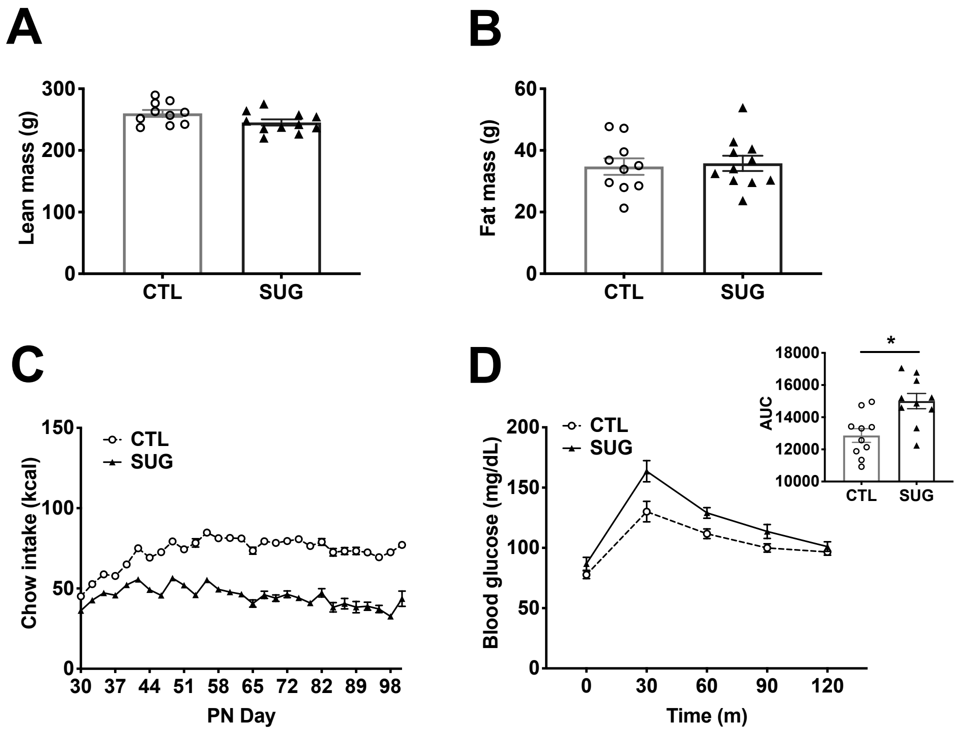

Figure S2: Effect of early life sugar consumption on food intake and metabolic measures (A, B) There were no differences in lean mass or in fat mass between animals fed sugar solutions or control animals (n=10,11; two-tailed, type 2 Student's T-test). (C) Kcals from chow intake were lower throughout the feeding period in animals fed early life sugar (n=10,11). (D) Results from the intraperitoneal glucose tolerance test show an elevated area under the curve (AUC) in rodents fed sugar solutions during early life (n=10,11; two-tailed, type 2 Student's T-test; $P<.05$). CTL=control, SUG= sugar, PN= post-natal day; data shown as mean $\pm$ SEM.

Figure S3

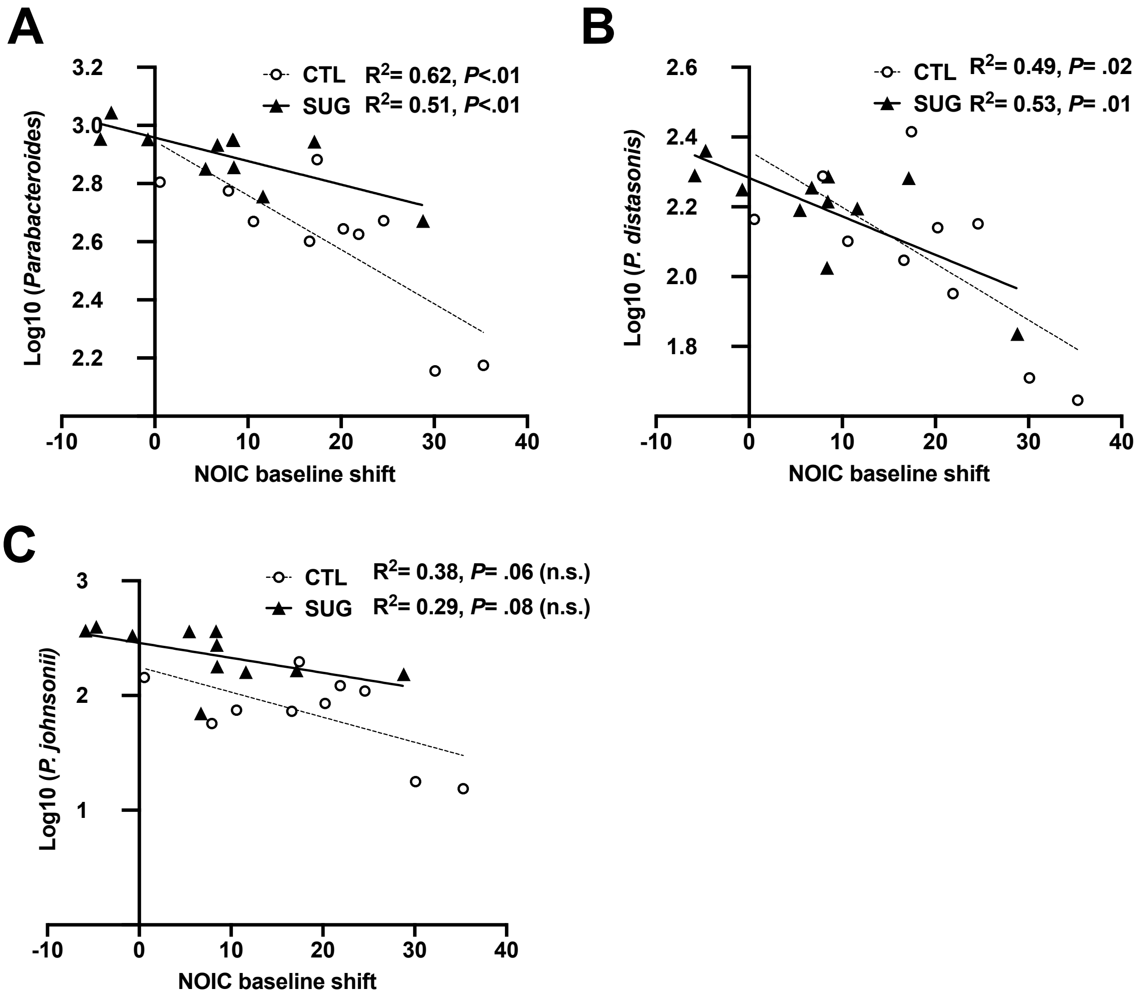

Figure S3: Relationship between *Parabacteroides* and behavioral outcomes in the Novel Object in Context task (NOIC) A) Linear regression of log normalized fecal *Parabacteroides* counts against shift from baseline performance scores in the NOIC task in sugar (SUG) and control (CTL) groups (n=10, 11). (B, C) Linear regression of the most abundant fecal *Parabacteroides* species against shift from baseline performance scores in NOIC across all groups tested (n=10, 11). *P<0.05; data shown as mean + SEM.

Figure S4

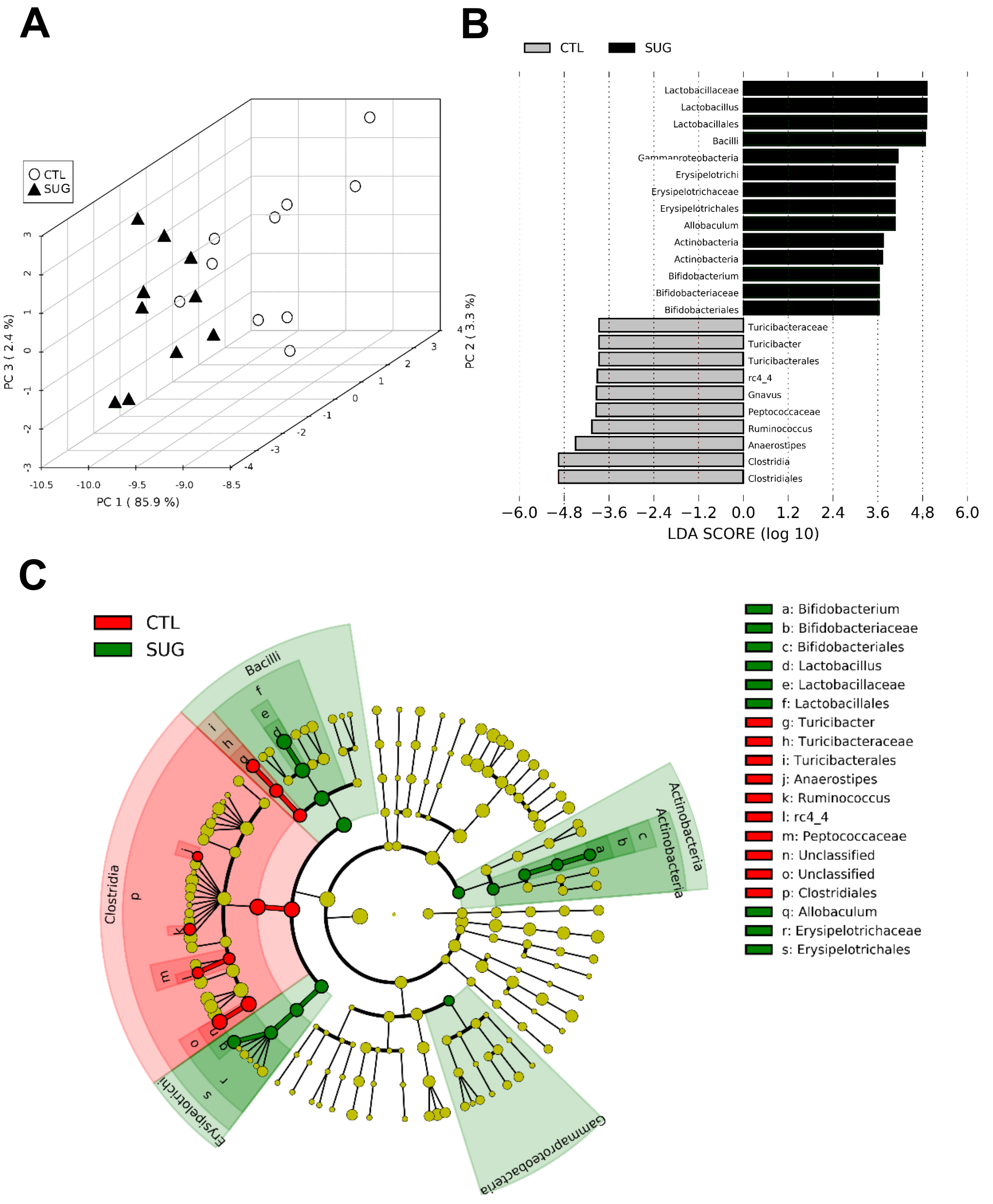

Figure S4: Effect of early life sugar consumption on the rat cecal microbiota (A) Principal component analysis (PCA) was run using all phylogenic levels (112 normalized taxa abundances) and shows different clustering patterns based on overall cecal microbial profiles.

(B) Linear discriminant analysis (LDA) Effect Size (LEfSe), run using the GALAXY platform, identified characteristic features of the cecal microbiota of rats fed a control diet or early life sugar. Relative differences among groups were used to rank the features with the LDA score set at 2. (C) Identified taxa are displayed by scores and on a phylogenic cladogram. CTL=control, SUG= sugar.

Figure S5

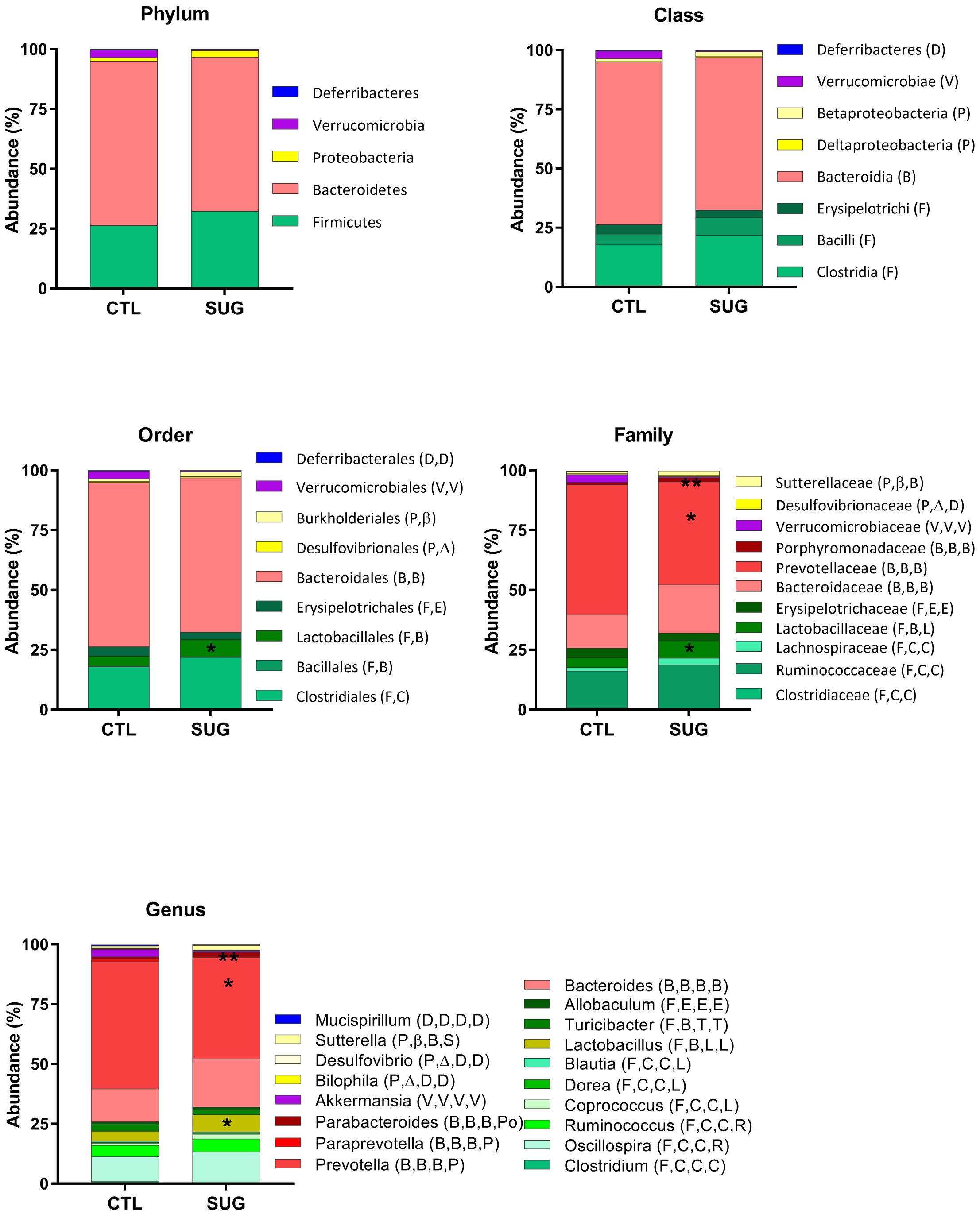

Figure S5: Effect of early life sugar consumption on the rat fecal microbiota.

Filtered bacterial abundances by taxonomic levels phylum, class, order, family, genus in fecal

samples from rats fed a control diets or early life sugar. Differences in abundances were assessed by Mann-Whitney non-parametric test. * $p<0.05$, *** $p<0.001$. CTL=control, SUG= sugar.

Figure S6

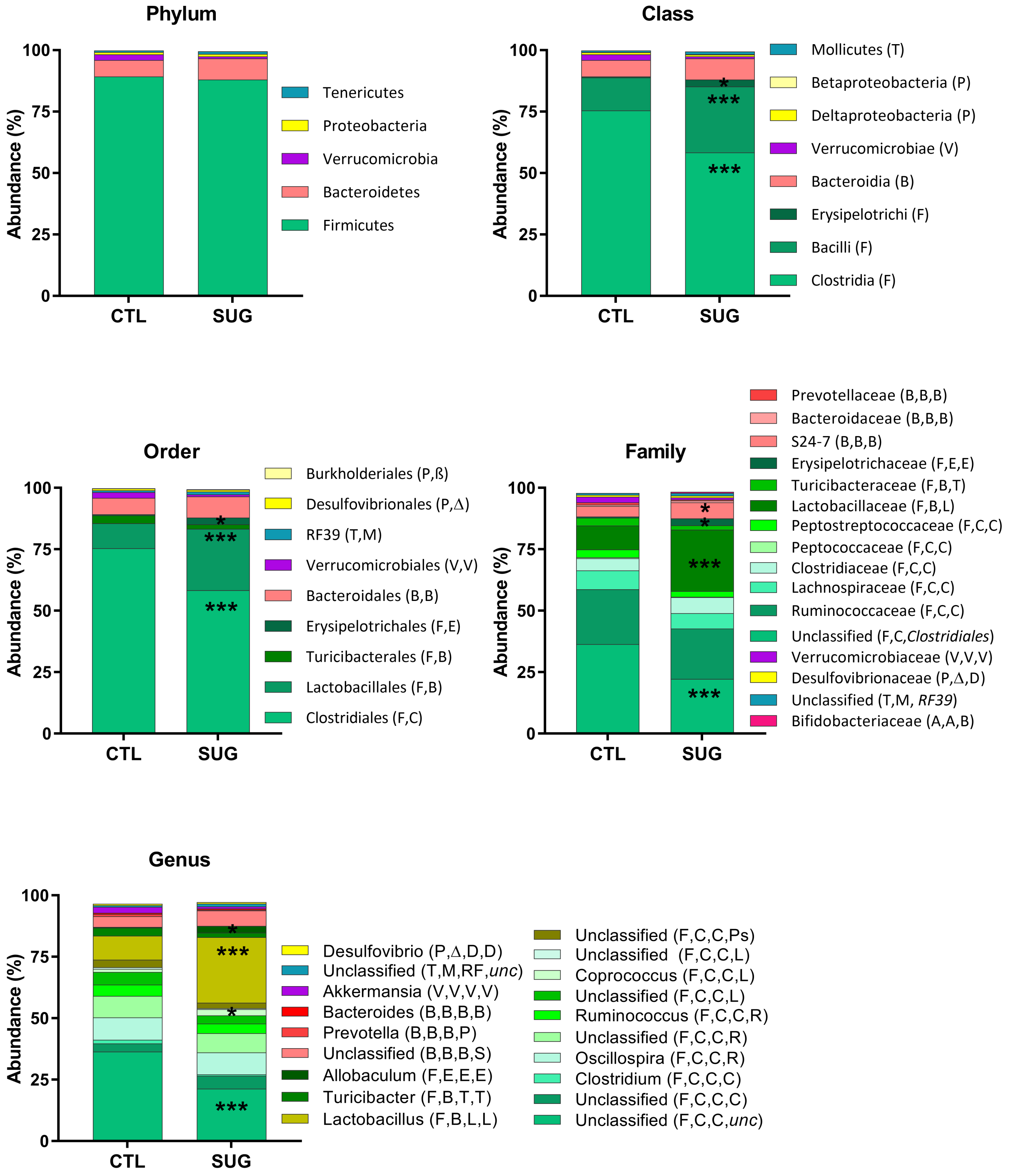

Figure S6: Effect of early life sugar consumption on the rat cecal microbiota:

Filtered bacterial abundances by taxonomic levels phylum, class, order, family, genus in cecal

samples from rats fed a control diets or early life sugar. Differences in abundances were assessed by Mann-Whitney non-parametric test. * $p<0.05$, *** $p<0.001$. CTL=control, SUG= sugar.

<u>Phylogenic taxonomy legend for Figure S5, S6:</u>

Phylum		Class		Order		Family		Genus
**F**	*Firmicutes*	**C**	*Clostridia*	**C**	*Clostridiales*	**C**	*Clostridiaceae*	*Clostridium*
						**L**	*Lachnospiraceae*	*Coprococcus*
								Blautia
								Dorea
						**P**	*Peptococcaceae*	
						**Ps**	*Peptostreptococcaceae*	
						**R**	*Ruminococcaceae*	*Oscillospira*
								Ruminococcus
		**B**	*Bacilli*	**L**	*Lactobacillales*	**L**	*Lactobacillaceae*	*Lactobacillus*
				**T**	*Turicibacterales*	**T**	*Turicibacteraceae*	*Turicibacter*
				**B**	*Bacillales*			
		**E**	*Erysipelotrichi*	**E**	*Erysipelotrichales*	**E**	*Erysipelotrichaceae*	*Allobaculum*
**B**	*Bacteroidetes*	**B**	*Bacteroidia*	**B**	*Bacteroidales*	**S**	*S24-7*	
						**B**	*Bacteroidaceae*	*Bacteroides*
						**P**	*Prevotellaceae*	*Prevotella*
								Paraprevotella
						**Po**	*Porphyromonadaceae*	*Parabacteroides*
**V**	*Verrucomicrobia*	**V**	*Verrucomicrobiae*	**V**	*Verrucomicrobiales*	**V**	*Verrucomicrobiaceae*	*Akkermansia*
**P**	*Proteobacteria*	**β**	*Betaproteobacteria*	**B**	*Burkholderiales*	**S**	*Sutterellaceae*	*Sutterella*
		**Δ**	*Deltaproteobacteria*	**D**	*Desulfovibrionales*	**D**	*Desulfovibrionaceae*	*Desulfovibrio*
								Bilophila
**T**	*Tenericutes*	**M**	*Mollicutes*	**RF**	*RF39*			
**D**	*Deferribacteres*	**D**	*Deferribacteres*	**D**	*Deferribacterales*	**D**	*Deferribacteraceae*	*Mucispirillum*
**A**	*Actinobacteria*	**A**	*Actinobacteria*	**B**	*Bifidobacter*	**B**	*Bifidobacteriaceae*	

Figure S7

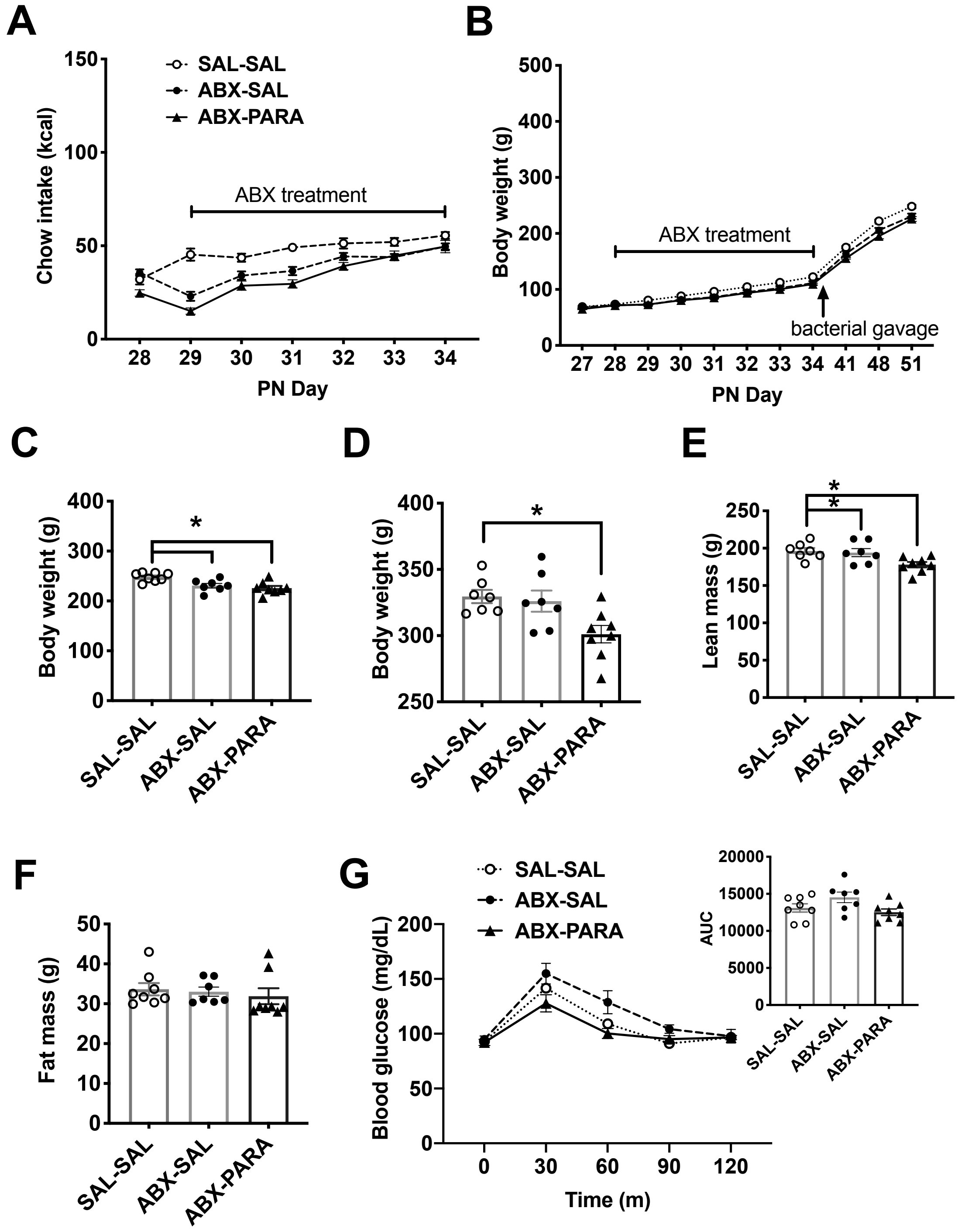

Figure S7: Experimental design, food intake, and metabolic measures for gut *Parabacteroides* enrichment (A) Effect of antibiotic treatment on food intake (B) and body weight (n=7,8). (C) Effect of gut *Parabacteroides* enrichment on body weight at PN 51 prior to

the start of behavioral testing (n=7,8; one way ANOVA with Tukey's post hoc test, $F_{(2,20)}$= 8.79; *P<.05) (D) Effect of gut *Parabacteroides* enrichment on body weight (n=7,8; one way ANOVA with Tukey's post hoc test, $F_{(2,19)}$= 5.7; *P<.05) (E) lean mass (n=7,8; one way ANOVA with Tukey's post hoc test, $F_{(2,19)}$= 5.33; *P<.05) (F) and body fat (one way ANOVA, n.s.) at PN 76. (G) Blood glucose levels during an interaperitoneal glucose tolerance test (IP GTT) (n=7,8 one way ANOVA for AUC; n.s.) SAL-SAL=saline-saline control, ABX-SAL= antibiotics-saline control, ABX-PARA= antibiotics-*P. johnsonii* and *P. distasonis* enriched, PN= post-natal day; data shown as mean $\pm$ SEM.

Figure S8

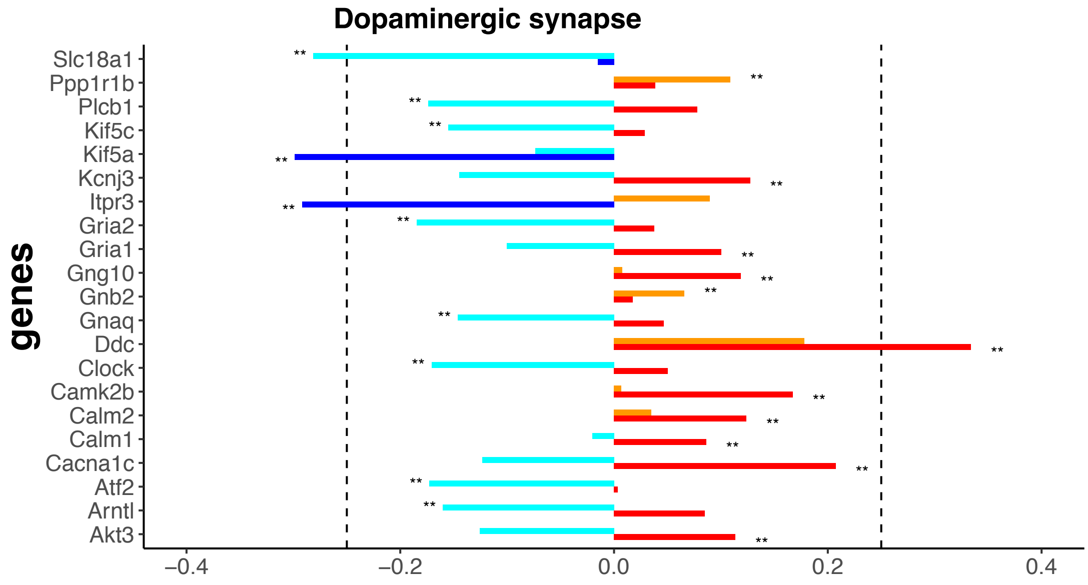

Figure S8: Comparison of hippocampal gene expression pathways altered by sugar and *Parabacteriodes*. The dopaminergic synapse pathway overlaps in the sugar and *Parabacteroides* transfer experiments. Red= upregulated by sugar, dark blue= downregulated by sugar, orange= upregulated by *Parabacteroides*, light blue= downregulated by *Parabacteroides*. * $P < 0.05$ and ** $P < 0.01$. Dotted line indicated ± 0.25 log2 fold change.
